# Brain hypoperfusion and nigrostriatal dopaminergic dysfunction in primary familial brain calcification caused by novel *MYORG* variants: case report

**DOI:** 10.1186/s12883-020-01910-1

**Published:** 2020-09-01

**Authors:** Shih-Ying Chen, Wei-Che Lin, Yung-Yee Chang, Tsu-Kung Lin, Min-Yu Lan

**Affiliations:** 1 Department of Neurology, Kaohsiung Chang Gung Memorial Hospital and Chang Gung University College of Medicine, 123 Ta-Pei Road, NiaoSong, Kaohsiung, 833 Taiwan; 2 Department of Diagnostic Radiology, Kaohsiung Chang Gung Memorial Hospital and Chang Gung University College of Medicine, 123 Ta-Pei Road, NiaoSong, Kaohsiung, 833 Taiwan; 3 Center for Parkinson’s disease, Kaohsiung Chang Gung Memorial Hospital and Chang Gung University College of Medicine, 123 Ta-Pei Road, NiaoSong, Kaohsiung, 833 Taiwan; 4 Center for Mitochondrial Research and Medicine, Kaohsiung Chang Gung Memorial Hospital and Chang Gung University College of Medicine, 123 Ta-Pei Road, NiaoSong, Kaohsiung, 833 Taiwan

**Keywords:** Primary familial brain calcification, Myogenesis regulating glycosidase, Dystonia, Parkinsonism, Cerebral hypoperfusion, Dopamine transporter

## Abstract

**Background:**

Primary familial brain calcification (PFBC) is a rare inherited disease characterized by multiple calcified foci in the brain parenchyma. *MYORG* is the first gene found to be associated with autosomal recessive PFBC. The precise pathogenic mechanism of neurodegeneration in PFBC remains unclear. The clinical phenotypes of PFBC are variable, and there is no clear correlation between clinical manifestations and radiological and pathological features of calcification.

**Case presentation:**

Two sisters in a Taiwanese family presented with young-onset Parkinsonism and multifocal dystonia. Their brain CTs showed multiple intracerebral calcifications. The genetic study detected two heterozygous novel variants, c.104 T > A (p.Met35Lys) and c.850 T > C (p.Cys284Arg) in the *MYORG* gene. In both patients, MR susceptibility weighted images revealed calcification of the deep medullary veins. Tc^99m^ ECD SPECT demonstrated a significant decrease of tracer uptake in the brain cortex and subcortical gray matter. Tc^99m^ TRODAT-1 SPECT revealed decreased tracer uptake in the bilateral striatum.

**Conclusion:**

Two novel *MYORG* variants were identified in Taiwanese family members presenting with PFBC. Abnormalities in the brain perfusion and dopamine transporter SPECTs suggest that cerebral ischemia due to extensive calcified vasculopathy, disruption of the basal ganglia-thalamo-cortical circuit, and nigrostriatal dopaminergic dysfunction are plausible pathogenic mechanisms of neurodegeneration in PFBC patients. Further investigation into the correlations between the pathogenicity-implicated imaging findings and the clinical phenotype are recommended.

## Background

Primary familial brain calcification (PFBC), previously known as Fahr’s disease, is a rare inherited disease characterized by multiple, generally symmetric calcified foci in the brain parenchyma, with tendency for basal ganglia, thalamus, and cerebellar white matter. The clinical phenotypes of PFBC are variable, with movement disorders, cognitive impairment, and psychiatric symptoms as the most common manifestations, while a significant number of asymptomatic cases exist [1]. Of note, there is no clear correlation between clinical manifestations and the radiological and pathological features of calcification [1]. Furthermore, the pathogenic mechanism of neurodegeneration in PFBC patients is unclear. Two modes of inheritance of PFBC have been characterized. Variants in *SLC20A2*, *XPR1*, *PDGFB*, and *PDGFRB* are associated with autosomal dominant PFBC. More recently, the discovery of PFBC-related bi-allelic variants of *MYORG* and *JAM2* have revealed the autosomal recessive aspect of the disease [2, 3]. We herein report on two novel *MYORG* variants causing PFBC in two Taiwanese family members. We also demonstrate cerebral blood perfusion abnormalities and dopaminergic dysfunction with radionucleid imaging modalities in the patients, indicating potential pathogenic mechanisms of neurodegeneration in PFBC patients.

## Case presentation

**Fig. 1:**
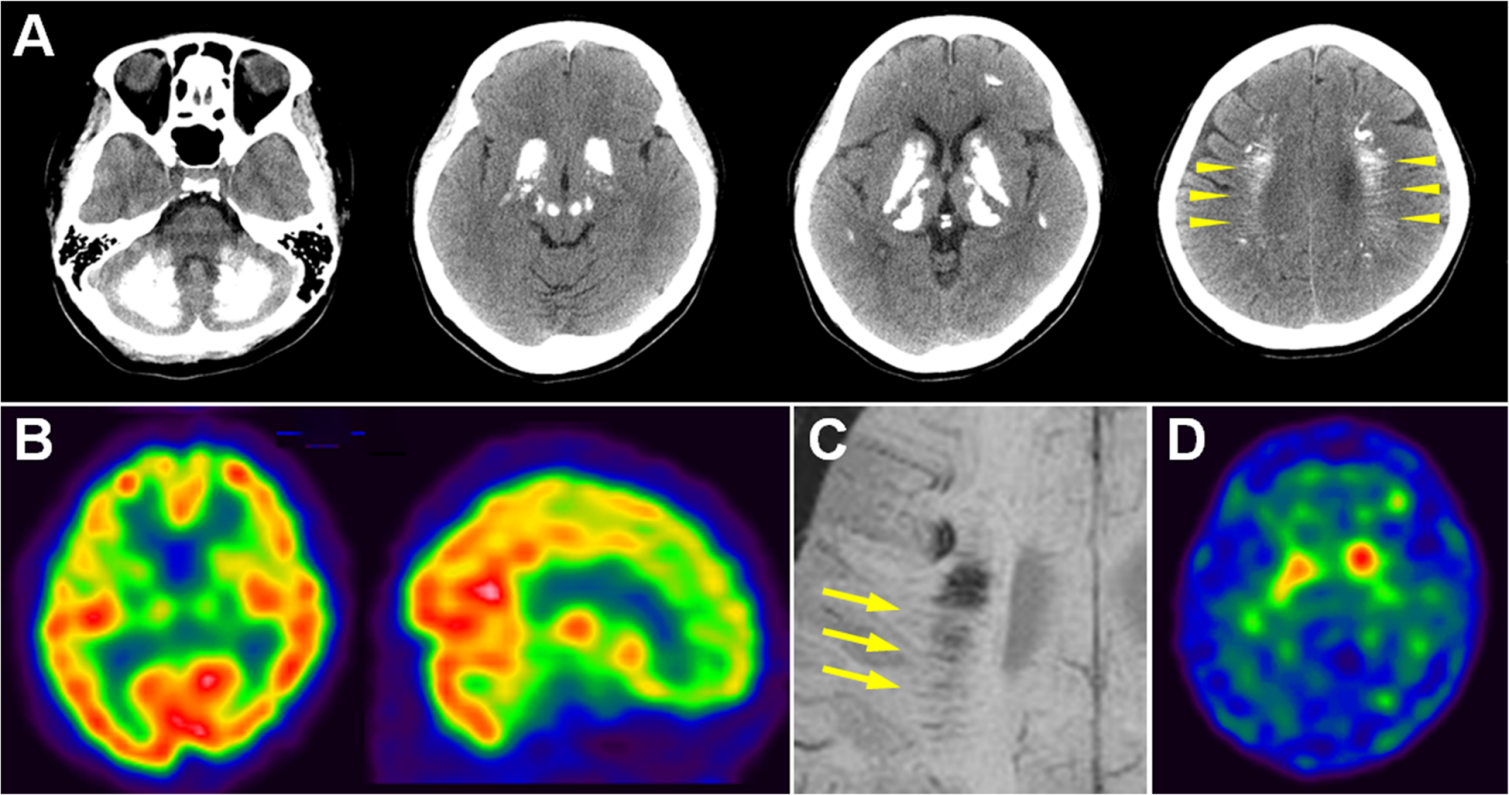
Brain images of Patient 1. **a** Brain CT shows severe calcification of bilateral basal ganglion, thalamus, caudate nucleus, red nucleus, and deep and subcortical white matter. The linear subcortical hyperdense streaks represent calcified medullary veins (arrowheads). **b** Tc^99m^ ECD SPECT demonstrates decreased blood perfusion in the bilateral frontal lobe, basal ganglion, and thalamus. **c** Calcified deep medullary veins (arrows) are hypointense on minimum intensity projection algorithm of MR susceptibility-weighted images. **d** Tc^99m^ TRODAT-1 SPECT shows decreased tracer uptake in bilateral striatum, more severe on the right side

The clinical data and study results of the patients are summarized in Table 1. The proband (Patient 1) was a 45-year-old woman who had awkwardness and “dragging” of the right leg when walking from one year prior to the study. She also complained of slurred speech and handwriting abnormalities from six months prior to the study. She had urinary frequency sometimes but denied postural dizziness, syncope episode, palpitation, bladder control dysfunction, constipation, hyperhidrosis or hypohidrosis. Neurological examinations revealed normal cognition, attention, affect, ocular movements, cranial nerves, pyramidal system and sensory functions, but showed dysarthria, low limb-predominant bilateral bradykinesia and rigidity, dysdiadochokinesia and irregular postural tremor of the left hand, oromandibular dystonia, and right leg action dystonia. The Mini-Mental State Examination (MMSE) score was 30/30 (normal ≥24), and the Montreal Cognitive Assessment Test (MoCA) score was 27/30 (normal ≥26). The Patient Health Questionaire-9 (PHQ-9) score was 4, suggesting minimal depression. The Unified Parkinson’s Disease Rating Scale (UPDRS) motor score was 14/108. Brain CT showed symmetric calcification in the bilateral basal ganglion, thalamus, caudate nucleus, red nucleus, and deep and subcortical white matter (Fig. 1a). The Total Calcification Score [4], a systematized assessment for severity of brain calcification, was 59. Blood tests excluded acquired metabolic (parathyroid hormone, calcium, phosphate, lactate, and thyroid hormones), autoimmune (antinuclear antibody, SSA/SSB antibodies, and rheumatoid factor), infectious (RPR, toxoplasma antibody, and HIV antibody) and toxic (lead, mercury, cadmium, manganese, and arsenic) causes of brain calcification.

**Table 1:** Summary of clinical information and study results of the two PFBC patients

	Patient 1	Patient 2
Sex	female	female
AAO/AOI (years)	44 / 45	early 3rd decade / 34
Clinical presentation	dystonia, bradykinesia, mild cerebellar sign	dystonia, bradykinesia
MMSE	30	26
MoCA	27	24
PHQ-9	4	7
UPDRS motor score	14	21
Total calcification score	59	46
Brain Tc^99m^-EDC SPECT	hypoperfusion of bilateral frontal lobe, basal ganglion and thalamus, and left parietal lobe	hypoperfusion of bilateral frontal and temporal lobes, basal ganglion and thalamus, right parietal lobe, and left cerebellum
Brain TRODAT-1 SPECT striatum/occipital cortex ratio^a^ (right, left)	1.69, 1.79	1.60, 1.60

^a^ Normal value > 1.90 for the age

*Abbreviations*: *AAO* age at onset, *AOI* age on investigations, *MMSE* Mini-Mental State Examination, *MoCA* Montreal Cognitive Assessment Test, *PHQ-9* Patient Health Questionaire-9, *UPDRS* Unified Parkinson’s Disease Rating Scale

The proband’s 34-year-old sister (Patient 2), had presented with intermittent head turning from her early 20s, which progressed slowly with time and did not cause dysfunction of her daily life. She denied weakness, limb awkwardness, or trunk imbalance. She had chronic constipation, but had no history of syncope episode, palpitation or bladder dysfunction. Neurological examination revealed mild symmetric bradykinesia and rigidity, cervical dystonia, and oromandibular dystonia. No other neurological abnormalities were found on examination. CT revealed multiple symmetric intracerebral calcifications, but with less severity than the proband (supplementary Figure 1), with a Total Calcification Score 46. Blood tests excluded secondary causes of abnormal brain calcification. MMSE score was 26/30, MoCA score was 24/30, PHQ-9 score was 7 (mild depression), and UPDRS motor score was 21/108. The patients’ mother exhibited normal neurological examinations and brain CT. Their father had also not presented features of cognitive impairment or movement disorder, having died of oral cancer at 59 years old. Other siblings refused examinations. The patients received levodopa and trihexyphenidyl. The clinical observation revealed marked reduction in bradykinesia and tremor when levodopa was used to 200–300 mg and trihexyphenidyl 3 mg daily for both of them, but no response of dystonia to the treatment.

**Fig. 2:**
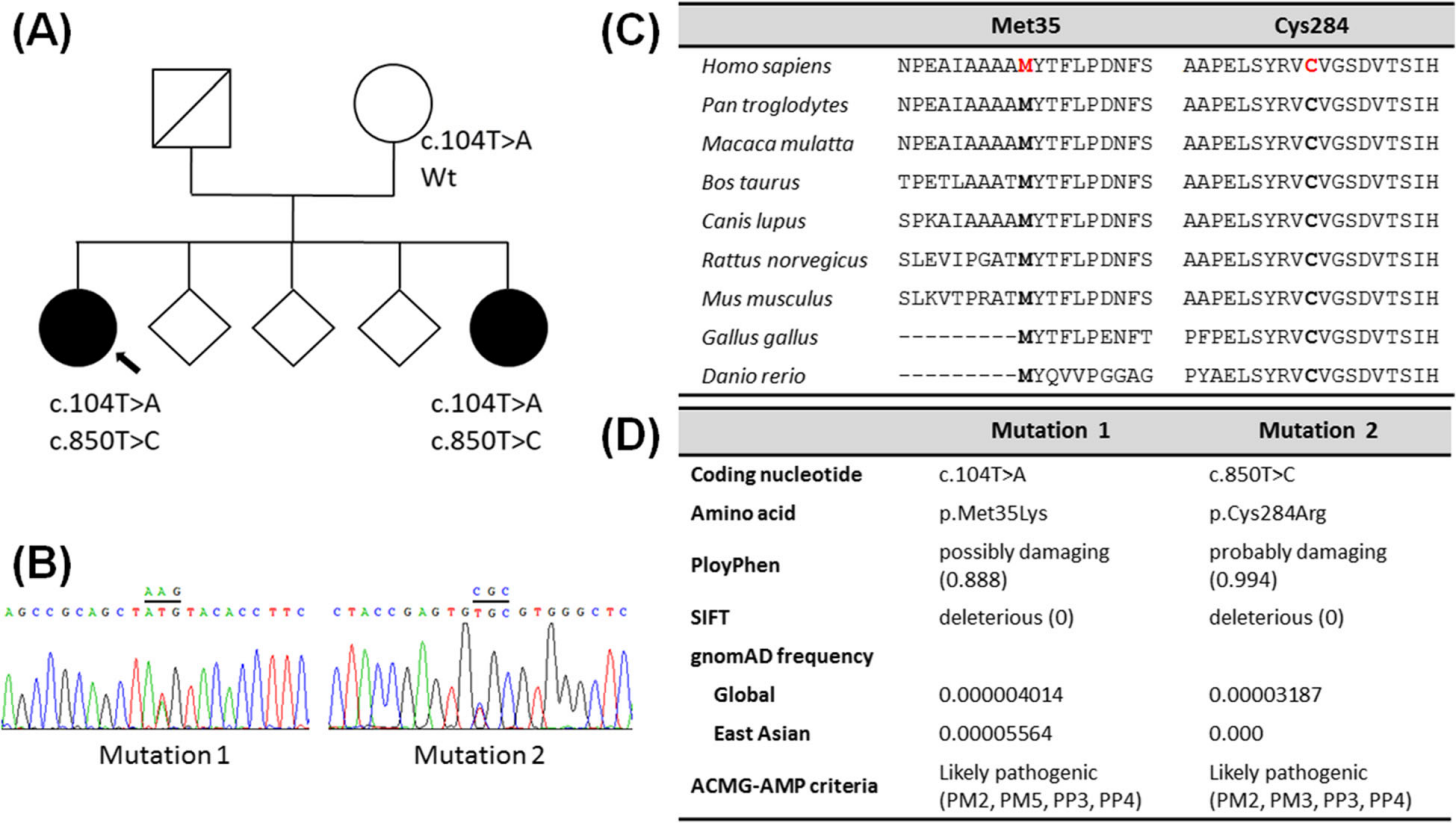
Pedigree and *MYORG* variants identified in the patient. **a** Family pedigree. Proband is indicated by arrow. Square represents male subject, circles represent female subjects, and diamonds represent the subjects whose gender was withheld for confidentiality reasons. Affected individuals are shown with solid symbols. Unaffected individuals or carriers are denoted with open symbols. Wt, wild type. **b** Electropherogram of the two identified *MYORG* variants. **c** Conservation of the variant residues across different species. **d** In-silico analysis and frequency in the populations of the two missense variants. ACMG-AMP, American College of Medical Genetics and Genomics and Association of Molecular Pathology

Given the characteristic brain CT findings and family history, a diagnosis of PFBC was considered plausible, and genetic study by whole exome sequencing was performed after obtaining informed consent. Capture of exome library was done using a SureSelect Human All Exon 50 Mb Kit (Agilent Technologies), and sequencing performed on an Illumina HiSeq2000 (Illumina). Approximate of 54 million paired-reads were mapped to the human reference genome. Variants detected in the known PFBC-related genes were reviewed. Two novel heterozygous variants, c.104 T > A (p.Met35Lys) and c.850 T > C (p.Cys284Arg) in the *MYORG* gene were detected and confirmed by Sanger sequencing (Fig. 2b). Both variants affected highly revolutionarily conserved amino acid residues, were predicted by in-silico analysis to be disruptive to protein function, and occur in extremely low frequencies in the general population according to the Genome Aggregation Database (Fig. 2c-d). A variant which also affects residue Met35, c.103A > G (p.Met35Val), has previously been reported in a PFBC kindred [2], suggesting the importance of the residue in maintaining functions of the encoded protein. Both of the two variants were classified as “likely pathogenic” based on the criteria of the American College of Medical Genetics and Genomics and the Association of Molecular Pathology on variant classification [5] (Fig. 2d). No pathogenic variants were detected in *SLC20A2*, *XPR1*, *PDGFB*, *PDGFRB*, or *JAM2.* The proband’s affected sister was also heterozygous for the two *MYORG* variants, and the mother carried a heterozygous c.104 T > A variant.

Cerebral blood perfusion in Patient 1 was investigated with Tc^99m^ ECD SPECT, which showed hypoperfusion of the bilateral frontal lobes, basal ganglion, thalamus, and right parietal lobe (Fig. 1b). The MR susceptibility weighted images (SWI) demonstrated that the deep medullary veins were hypointense on minimum intensity projection algorithm (Fig. 1c, arrows). The SWI finding is consistent with calcification of the deep medullary veins, as the parallel hyperdense streaks shown on CT (Fig. 1a, arrowheads). To elucidate the mechanism underlying the patient’s extrapyramidal signs, Tc^99m^ TRODAT-1 SPECT was performed to assess nigrostriatal dopaminergic function. It exhibited decreased tracer uptake in the bilateral striatum (stratum/occipital cortex ratio, right side 1.69, left side 1.75, normal > 1.90) (Fig. 1d). Brain blood perfusion SPECT, dopamine transporter SPECT, and MR SWI showed similar changes in Patient 2 (Table 1, supplementary Figure 1).

## Discussion and conclusion

Myogenesis regulating glycosidase (MYORG), the protein encoded by *MYORG*, is a lamina-associated nuclear envelope transmembrane protein belonging to the glycosidase family 31. Expression study has previously revealed that *MYORG* is specifically expressed in the astrocytes [2]. It is postulated that MYORG regulates protein glycosylation in the endoplasmic reticulum of astrocytes, and PFBC caused by *MYORG* mutation is related with astrocyte dysfunction [2]. Furthermore, astrocytes and other PFBC protein-expressing cells including vascular smooth muscle cells, periocytes, and endothelial cells comprise the neurovascular unit. Pathologically, calcium-phosphate deposition initiates in or around cerebral blood vessels in PFBC patients and mouse models [6–8]. Dysfunction of the neurovascular unit may thus be the molecular basis which contributes to the notable calcium deposition in PFBC.

Calcium deposits in PFBC are most frequently located in the basal ganglion, subcortical white matter, cerebellum, and thalamus, irrespective of causative genes. Brainstem, more specifically pons, calcification is considered highly suggestive of *MYORG* mutations [9]. In our patients, neuroimaging findings were remarkable for widespread symmetric calcifications, also including the midbrain (red nucleus) in Patient 1. Clinically, akinetic-dystonic extrapyramidal signs, depression and frontal executive dysfunction are the most common phenotypes for PFBC [1, 4]. The major clinical features of our patients were movement disorders including Parkinsonism and dystonia. Patient 1 also presented with speech disturbance which is very common (85%) for patients with *MYORG* alternations [1]. Patient 2 had mild cognitive impairment characterized by visuospatial and executive dysfunction and mild depression, as revealed by MoCA and PHQ-9.

The exact pathogenic mechanism of neurodegeneration in PFBC remains unclear. Of note, there is no evident link between the severity or distribution of calcium deposition and clinical manifestations [1]. In our patients, radionuclide images revealed hypoperfusion in the basal ganglion and thalamus, as well as in the frontoparietal cortex, corresponding to the clinical phenotypes characterized by dysfunction of frontal lobes and basal ganglion [10]. Calcification of the deep medullary veins in the centrum semiovale was clearly shown on MR SWI. To our knowledge, there is currently no reported neuropathological examination of patients with PFBC related to *MYORG* mutations. However, pathological study in autosomal dominant PFBC has demonstrated severe cerebrovascular impairment, as evidenced by varying degrees of calcification in blood vessels of different sizes and obstruction of the lumen in severely affected vessels [7]. The process initiates with deposition of tiny calcium spheroids on vessel walls of otherwise normal capillaries, and involves broad regions of the brain, including the basal ganglia, thalamus, cerebellar white matter as well as regions at a considerable distance from the main calcification foci [6, 11]. Indeed, ischemic stroke with deep cerebral infarction has been reported in two patients with PFBC who did not exhibit vascular risk factors [12, 13]. Aside from ischemia, deafferentation of regional neurons may cause decreased tracer uptake in brain blood perfusion SPECT [10]. There exist distinct neural circuits centered on the basal ganglia and connecting with the substantia nigra, thalamus, cerebellum, frontal lobe and other brain regions [14]. These circuits are essential for motor and high level behavioral controls. Neuronal dysfunction in the basal ganglia due to calcification may disrupt its connection with, for instance, the frontal cortex through the basal ganglia-thalamo-cortical circuit and lead to hypoperfusion of the deafferent brain cortex. In addition, a decrease of radionuclide tracer uptake by dopamine transporters in the striatum was also noted in our patients, indicating dysfunction of the nigrostriatal dopamine pathway. Impairment of the nigrostriatal pathway has previously been demonstrated in PFBC patients who clinically presented with Parkinsonian features, caused by *SLC20A2* and *XPR1* mutations [15, 16]. Neuronal loss with Lewy bodies has been evidenced in the substantia nigra and other brain regions in the neuropathology of patients with *SLC20A2* mutations [7, 17]. However, this pathological change is not presented in all PFBC patients, and it is unclear whether this finding is merely coincidental. Taken collectively, these findings suggest that cerebral ischemia due to extensive calcified vasculopathy, disruption of the basal ganglia-thalamo-cortical circuit, and nigrostriatal dopaminergic dysfunction are plausible pathogenic mechanisms of neurodegeneration in PFBC patients. We recommend further investigation to clarify the correlation between the pathogenicity-implicated imaging findings and the clinical phenotypes.

In conclusion, we identified two novel variants in *MYORG* responsible for PFBC. Imaging studies of the patients showed a decrease of regional cerebral blood perfusion and impairment of the nigrostriatal dopamine pathway, indicating potential pathogenic mechanisms underlying neurodegeneration in PFBC patients.

## Supplementary information

**Additional file 1: Supplementary Figure 1**. Brain images of Patient 2. (A) Brain CT shows calcification of bilateral basal ganglion, thalamus, caudate nucleus, and cerebral white matter. (B) Tc^99m^ ECD SPECT shows decreased blood perfusion in the bilateral frontal and temporal lobes, basal ganglion and thalamus, right parietal lobe and left cerebellum. (C) Calcified deep medullary veins (arrows) are demonstrated on minimum intensity projection algorithm of MR susceptibility-weighted images. (D) Tc^99m^ TRODAT-1 SPECT shows decreased tracer uptake in bilateral striatum.

## Data Availability

All data generated or analyzed during this study are included in this published article and its supplementary information files.

## Abbreviations

MMSE: Mini-Mental State Examination
MoCA: Montreal Cognitive Assessment Test
MYORG: myogenesis regulating glycosidase
PFBC: primary familial brain calcification
PHQ-9: Patient Health Questionaire-9
SWI: susceptibility weighted images
UPDRS: Unified Parkinson’s Disease Rating Scale
