# Supplementary figures and images for: Brain hypoperfusion and nigrostriatal dopaminergic dysfunction in primary familial brain calcification caused by novel MYORG variants: case report
Source: BMC Neurol. 2020 Sep 1;20:329. doi: 10.1186/s12883-020-01910-1 (PMC7460774; doi:10.1186/s12883-020-01910-1)

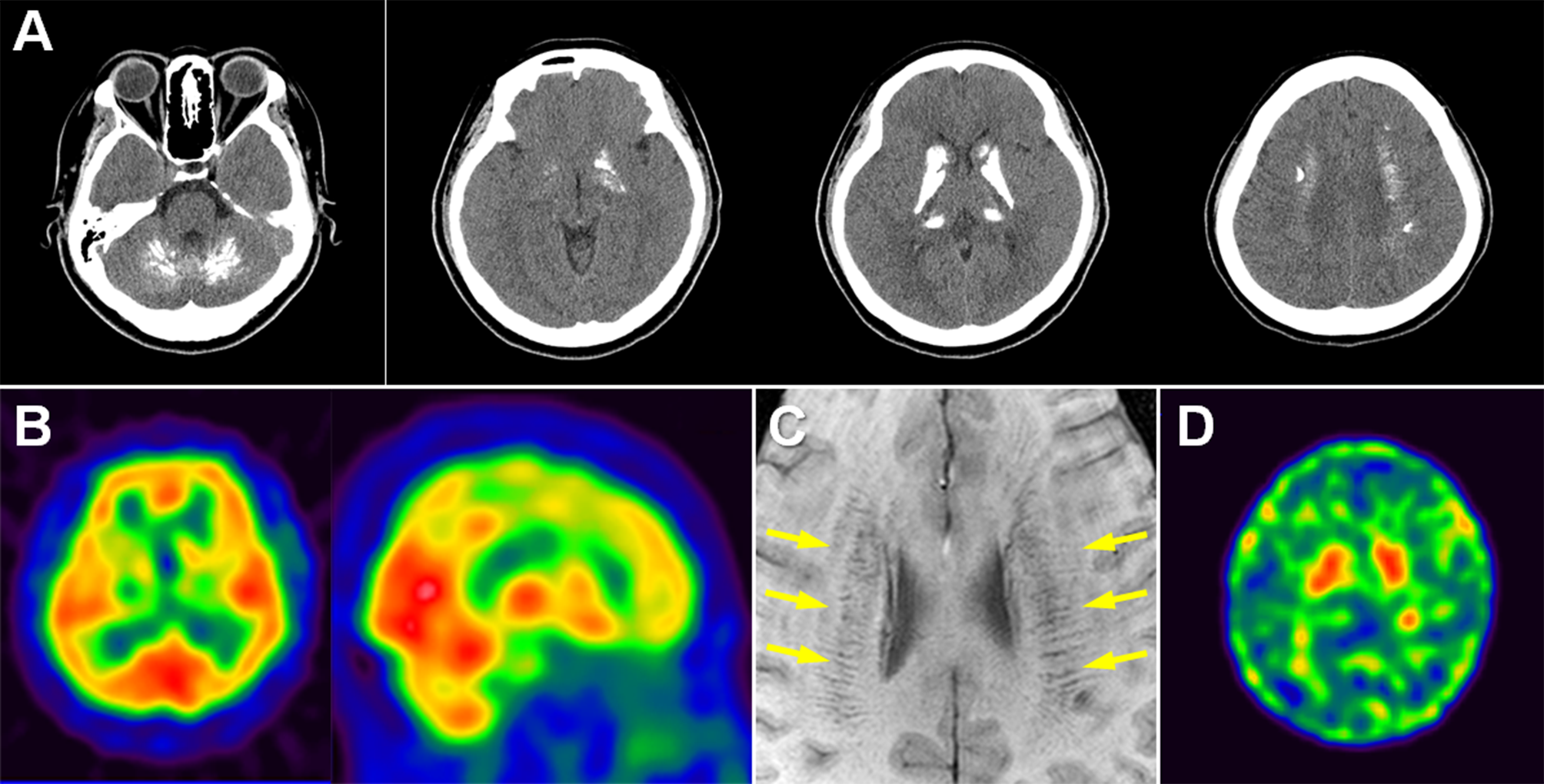

Supplement: Supplementary file 1 — Additional file 1: Supplementary Figure 1. Brain images of Patient 2. (A) Brain CT shows calcification of bilateral basal ganglion, thalamus, caudate nucleus, and cerebral white matter. (B) Tc99m ECD SPECT shows decreased blood perfusion in the bilateral frontal and temporal lobes, basal ganglion and thalamus, right parietal lobe and left cerebellum. (C) Calcified deep medullary veins (arrows) are demonstrated on minimum intensity projection algorithm of MR susceptibility-weighted images. (D) Tc99m TRODAT-1 SPECT shows decreased tracer uptake in bilateral striatum. [file 12883_2020_1910_MOESM1_ESM.tif]
